# Structural Evolution of Nanoscale Zero-Valent Iron (nZVI) in Anoxic Co^2+^ Solution: Interactional Performance and Mechanism

**DOI:** 10.1038/srep13966

**Published:** 2015-09-10

**Authors:** Yalei Zhang, Wen Chen, Chaomeng Dai, Chuanlong Zhou, Xuefei Zhou

**Affiliations:** 1 State Key Laboratory of Pollution Control and Resources Reuse, Tongji University, Shanghai 200092, China; 2 College of Civil Engineering, Tongji University, Shanghai 200092, China

## Abstract

The structures of nanoscale zero-valent iron (nZVI) particles evolving during reactions, and the reactions are influenced by the evolved structures. To understand the removal process in detail, it is important to investigate the relationships between the reactions and structural evolution. Using high resolution-transmission electron microscopy (HR-TEM), typical evolved structures (sheet coprecipitation and cavity corrosion) of nZVI in anoxic Co^2+^ solutions were revealed. The system pH (pH measured in mixture), which controls the stability of coprecipitation and the nZVI corrosion rate, were found to be the determining factors of structural evolutions. X-ray photoelectron spectroscopy (XPS) results indicated that the formation and dissolution of sheet structure impacts on the ratio of Fe(0) on the nZVI surface and the surface Co^2+^ reduction. The cavity structure provides the possibility of Co migration from the surface to the bulk of nZVI, leading to continuous removal. Subacidity conditions could accelerate the evolution and improve the removal; the results of structurally controlled reactions further indicated that the removal was suspended by the sheet structure and enhanced by cavity structure. The results and discussion in this paper revealed the “structural influence” crucial for the full and dynamical understanding of nZVI reactions.

Nano zero-valent iron (nZVI) has been widely investigated as an effective and environment-friendly material^1^,^2^,^3^,^4^,^5^. The nanoscale particle size and low oxidation reduction potential make nZVI an efficient sorbent and reductant^5^,^6^ for treating many types of organic contaminants^7^,^8^,^9^,^10^, including chlorinated methane, organochlorine pesticides, chlorinated phenols, organic dyes, and most heavy metals^11^,^12^,^13^,^14^,^15^,^16^,^17^,^18^,^19^,^20^,^21^,^22^,^23^,^24^, such as Ag^2+^, As(III), Ba^2+^, Cd^2+^, Co^2+^, Cr (V), Cu^2+^, Ni^2+^, Pb^2+^, and Zn^2+^.

The mechanisms of contaminant sequestration by nZVI have been studied and are mostly attributed to surface reduction and complexation^1^,^2^,^3^,^10^,^11^,^12^. However, the removal process can also be affected by structural transformation of nZVI. First, nZVI was reported to be unstable, with continuous structural changes during the reactions; the distinct structural nZVI particle transformations observed during contaminant removal can be classified into two categories: accretion on nZVI surface due to the formation of hydroxide or coprecipitation^13^,^22^,^24^,^25^, and corrosion of nZVI core due to reactions with contaminants, water, oxygen and other oxidants^1^,^13^,^14^. Secondly, the transformed structures significantly affect nZVI reactions: the formation of oxides on the nZVI surface makes removal slower and less efficient^11^,^18^,^22^, and the corrosion of nZVI core can affect the migration of contaminants on nZVI particles^13^. Finally, the structural transformation and its effects are dynamic in some reactions: the structure undergoes a series of transformations and evolves over the course a long term reaction, leading to a sequence of effects, which not only alter the removal properties of nZVI but also give rise to further structural transformations^1^,^13^,^26^. However, researchers have always described the removal performance and the structural transformations statically and separately with few descriptions of structural evolutions; furthermore, most structural transformations were only regarded as consequences of removal, and no studies have combined investigations of reaction and structural evolution in the removal process and mechanism.

To elucidate the evolution process and its effects in detail, Co^2+^ solution was chosen as the target contaminant because it can provide a potential long-term reaction with nZVI due to the small oxidation reduction potential difference between cobalt and iron (Co → Co^2+^ + 2e^−^, E_0_ = −0.28 V, Fe → Fe^2+^ + 2e^−^, E_0_ = −0.41 V). Additionally, cobalt is one of environmentally hazardous heavy metals that is mainly introduced into the environment by its use in medicines, paints, pigments, and metallurgical, mechanical and electronic applications. Long term exposure to cobalt can be increasing hazardous to human beings, with effects such as paralysis, diarrhea, lung irritations, heart failure, bone defects and even cancer^27^,^28^,^29^.

In this work, reactions between nZVI and Co^2+^ at different initial concentrations in anoxic aqueous solutions (to eliminate the effects of O_2_) were tracked for 10 days using a variety of methods including inductively coupled plasma optical emission spectrometry (ICP-OES), high resolution-transmission electron microscopy (HR-TEM), energy dispersive spectroscopy (EDS), X-ray photoelectron spectroscopy (XPS), and scanning electron microscopy (SEM). Continuous removal and reduction of Co^2+^ by nZVI caused by structural evolution were revealed in reaction processes. The evolved structure, evolution process and effect of pH were discussed in detail. Experiments controlled by system pH and nZVI structure were conducted to augment these results. Moreover, “structural influence” was proposed and used to explain the controversial results obtained by previous studies.

## Results and Discussion

### Removal Results

The results of Co^2+^ removal by nZVI in the short term and long term are shown in Figure S1-S4 (“S” indicates figure and table in supporting information). The removal proceeded over a period of 10 days, and the highest achieved removal capacity was 452.2 mg/g. Details of the results of the short term and long term experiments and comparisons with the results of previous research are presented in SI Section 2. It should be noted that, as previously reported, equilibriums are achieved in a very short time of within 1 hour^18^; however, long term reactions altered the equilibriums and led to a constant removal. Kinetics fitting analyses of short term and long term removal are presented in SI Section 3. The continuous release of Fe^2+^ over 10 days also indicated a further reaction between nZVI and Co^2+^.

### Structure Evolution

**Figure 1:**
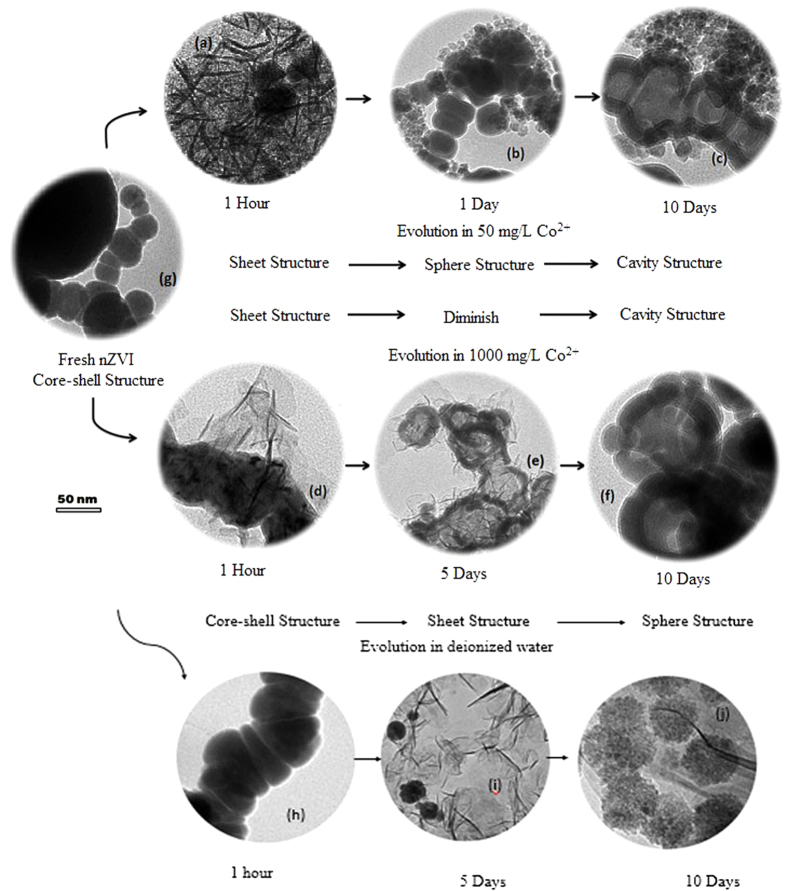
Typical TEM images of structural evolution of 1 g/L nZVI particles reacting with deionized water and with Co^2+^ at different initial concentrations over 10 days.

Figure 1 shows images of typical structural evolution of nZVI during 10 days’ reaction with deionized water and with Co^2+^ at initial concentrations of 50 and 1000 mg/L. Distinct structural evolution of nZVI particles was observed. The fresh nZVI particles are shown in Fig. 1g (Figure S5) and exhibit a chain-like core-shell structure in agreement with previously reports^18^,^30^. In the reaction with 50 mg/L of Co^2+^, particles with sheet wrapping structures were formed after 1 hour (Fig. 1a, Figure S6); the sheet structures were then transformed to numerous sphere structures (Fig. 1b, Figure S7) after 1 day’s reaction, and finally were converted to the chain-like cavity structures (Fig. 1c, Figure S8) surrounded by sphere structures. In the reaction with 1000 mg/L of Co^2+^, chain-like structures with sheet wrapping were formed after 1 hour (Fig. 1d, Figure S9); the cores were then hollowed and the sheet structures gradually diminished (Fig. 1e, Figure S10); finally the structure evolved to pure cavity agglomeration (Fig. 1f, Figure S11). The surface evolutions were distinctly different in different Co^2+^ concentrations, however, cavity structures were obtained for both cases, implying a tendency to inner corrosion. In the reaction with deionized water, particles still existed after 1 hour (Fig. 1h, Figure S13); the sheet structures (Fig. 1i, Figure S14) were then formed after 5 days’ reaction, and finally were converted to the numerous sphere structures (Fig. 1j, Figure S15).

**Figure 2:**
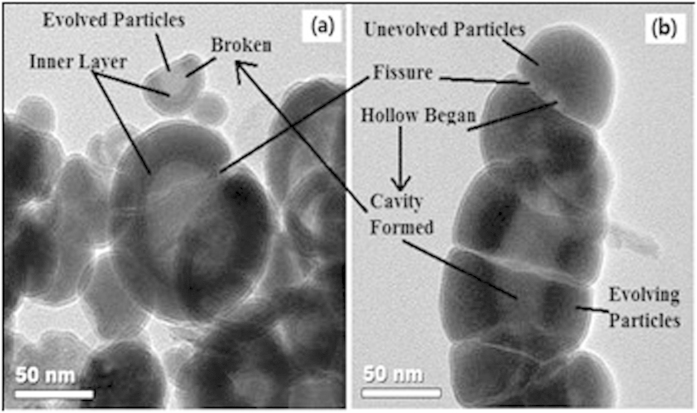
TEM images of cavity structures after reaction in 1000 mg/L Co^2+^ for 10 days.

The EDS analysis results (Figure S16) show that the core structures consist mainly of Fe metal; the sheet structures mainly contain iron and cobalt hydroxide; the sphere structures mainly contain iron hydroxide; the cavity structures may contain Fe and Co metals. That is to say, the Co content was enriched on accretion and corrosion structures. It also should be noted that the cavity structure may not be a closed sphere but more similar to a bowl or a hollowed sphere as will be discussed in detail below (Fig. 2, Figure S11).

### Sheet Structure

**Figure 3:**
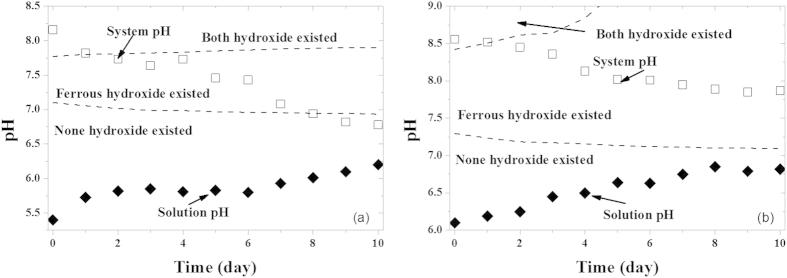
Variation of system pH and solution pH at different initial Co^2+^ concentrations ((a) 1000 mg/L; (b) 50 mg/L).

The formation and dissolution of sheet structure could be associate with the variations of system and solution pH values (pH measured in the centrifuged solution) shown in Fig. 3. Theoretically, no hydroxide could exist in an aqueous solution whit a pH lower than the value shown by the line in the bottom of Fig. 3; both Fe(OH)_2_ and Co(OH)_2_ can exist when pH is higher than the value shown by the top line in Fig. 3; only Fe(OH)_2_ can exist when pH is between the two values (the calculation procedure is presented in SI Section 7). Initially, coprecipitation (Fig. 1a,d) was observed in both systems due to the high system pH above the top line; the Co hydroxides then gradually dissolved when the system pH decreased below the top line, resulting in the decreased number of the sheet structure in 1000 mg/L Co^2+^ solution (Fig. 1e) and the transformation of the sheet structure to the sphere structure in 50 mg/L Co^2+^ solution (Fig. 1b); finally, the hydroxides in 1000 mg/L Co^2+^ solution completely dissolved (Fig. 1f) because the system pH was below the bottom line (Fig. 3a), whereas, the hydroxides in 50 mg/L Co^2+^ solution partly remained (Fig. 1c) because the system pH was between the two lines (Fig. 3b). Fresh nZVI (Fig. 1g) and nZVI stored in deionized water after 1 h (Fig. 1h) had the same core-shell structure; the Fe hydroxides then gradually dissolved when the system pH decreased below the bottom line, resulting in the decreased number of the sheet structure in deionized water (Fig. 1i) and the transformation of the sheet structure to the sphere structure (Fig. 1j).

The nZVI solution system is heterogeneous and is comprised of a solid phase (higher pH) and a liquid phase (lower pH). The sheet structure formed on the solid phase, which exhibits a higher pH. However, the rapid formation of the sheet structure cannot be related only with pH. The interface of nZVI was first dominated by the solid phase, i.e., zero valent iron, which has a low standard potential; the electrons could rapidly been transported to the acceptor (water and Co^2+^), leading to excessive Fe^2+^ release; in colloidal system, supersaturation of the interface would make the cationic concentration in the immediate vicinity (FeOOH shell) of the solid particles usually higher than the bulk concentration, leading to precipitate formation at lower pH of the liquid phase. All of these effects make the formation of sheet structure an unstable and excessive process. However, when the sheet structure became the interface, the rapid electrons transfer between nZVI and solution was stopped by the sheet structure with the higher standard potential such that no excessive Fe^2+^ was released, and the liquid phrase with the lower pH became dominant, leading to the gradual dissolution of the sheet structure.

### Cavity Structure

The formation of cavity structure was widely observed in previous studies^13^,^25^ and this corrosion of the iron core is also sensitive to the changes in pH. Similar structures could be obtained in similar pH conditions. Figure S17 shows structures observed at different pH values of the system for 1 g/L nZVI added into 50 mg/L Co^2+^, and the system pH kept at 8.5, 7.5, 6.5 and 5.5, respectively. No cavity structures were formed at a high system pH of 8.5 (Figure S20a), similar to the sheet structures shown in Fig. 1a observed at the system pH of 8.59. A sheet wrapped cavity structure (Figure S20b) corresponds to Fig. 1e, which was obtained at the system pH of 7.64 after reacting with 1000 mg/L Co^2+^ for 3 days. Pure cavity structures (Figure S20c) correspond to Fig. 1f, which was formed at system pH of 6.78 after reacting with 1000 mg/L Co^2+^ for 10 days. Lower system pH (5.5) values could lead to the presence of the cavity with thinner walls (Figure S20d). Obviously, lower system pH could accelerate the corrosion of nZVI and the formation of the cavity structure.

The evolution of a particular nZVI particle should be different because the actual reaction condition of every nZVI particle could be quite different even for the same reaction time. Three stages could be defined for the formation of cavity structures as shown in Fig. 2 obtained after reaction in 1000 mg/L Co^2+^ over 10 days. The unevolved particles have integrated core-shell structure; evolving particles’ core was hollowing and chambers formed; finally the particles evolved to the more hollowed cavity and even broken cavity structures with an obvious inner layer. The corrosion may start at the fissure regions of the FeOOH shell or sheets which showed a thinner hydroxide layer; meanwhile, other regions of the particle could not be corroded because they were wrapped by the thicker hydroxide layer; then corrosion digs into the particles through the fissures forming cavity; finally the fissure became larger and the particle was broken, and an inner layer was formed to stop further corrosion. However, it is difficult to track and determine the evolution process of one particular nZVI particle.

### Surface Reduction

**Figure 4:**
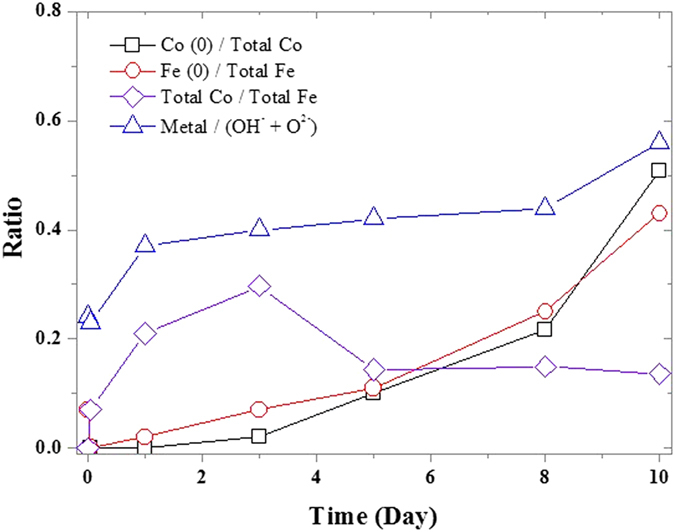
XPS peak area ratios of Co(0) to total Co, Fe(0) to total Fe, total Co to total Fe and metal to (OH^−^ + O^2−^) in 1000 mg/L Co^2+^.

XPS analysis was conducted to determine the compositional variations at the surface during the structural evolution (detailed survey of the XPS spectra is discussed in SI Section 6). Figure 4 presents the ratios of different elements on the nZVI surface at different reaction times in 1000 mg/L Co^2+^ by calculating the XPS peak areas. The ratio of Fe(0) to total Fe first decreased to zero due to the rapid formation of the sheet structure, and then gradually increased even surpassing that in the fresh nZVI due to the dissolution of sheet structure. The fraction of Co(0) increased up to 52% with the rise of Fe(0) fraction, indicating a further reduction by nZVI. The increasing ratio of metal to (OH^−^ + O^2−^) on the nZVI surface also indicated the increase of the zero valent metal content.

The surface reduction of Co cannot be determined by XPS in a short time period, as described by Uzum^18^; the electrons must go through the rapidly formed sheet structure, which has a higher standard potential than the metallic iron. However, when the obstruction (sheet structure) became sufficiently thin with the structural evolution, the electrons could transfer to the Co^2+^ ions which have a lower standard potential than the metallic iron, thus achieving the surface reduction of Co. It should be noted that the long term reaction exposed more Fe(0) on nZVI surface, which could also increase the opportunity of surface reduction. The possible reason could be that the long term reaction would destroy the original FeOOH shell, or that the formation of cavity could lead to the exposure of the bulk zero valent iron.

### Migration of Co

**Figure 5:**
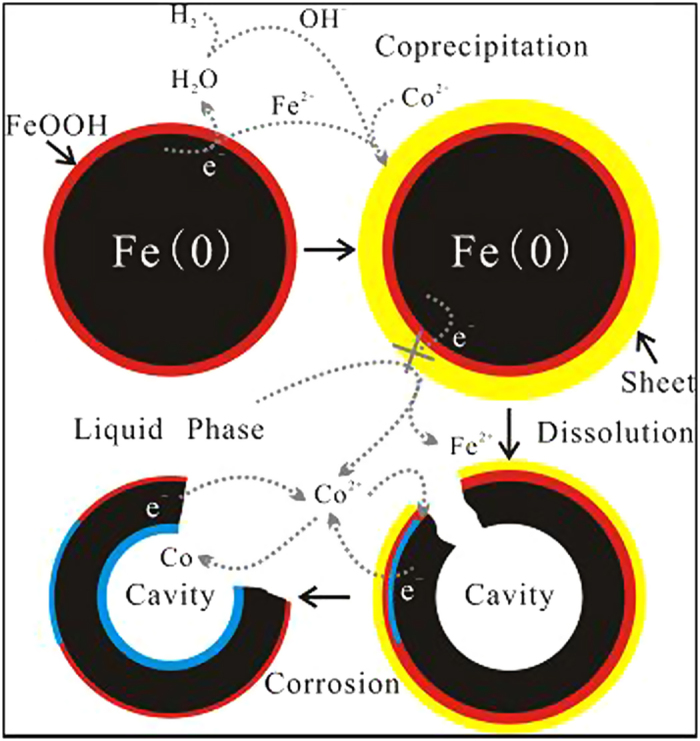
Conceptual process and mechanism of Co migration on nZVI particles.

As shown in Fig. 4, the ratio of total Co to total Fe was distinct. A peak appeared on the third day and the the ratio was constant after the fifth day. The results suggest that the Co content was enriched on the nZVI surface in the first three days and was subsequently released from the nZVI surface. This is also corroborated by the XPS analysis: the excursion of OH- peak on O 1s (Figure S19) indicates that the surface hydroxides lead to the transformation from FeOOH to Fe/Co coprecipitation on nZVI when the sheet structure was formed, and back to FeOOH when the sheet structure dissolved. However, Co^2+^ concentration in solution did not increase while Co atoms were released from the sheet structure; in other words, the excessive removed Co atoms did not enriched the Co content on the nZVI surface. EDS analysis (Figure S16) shows that Co could be detected on the sheet structure and the cavity structure but cannot be detected on the compact iron core. Therefore, the path of Co migration on nZVI particles could be deduced, as shown in Fig. 5: Co atoms were firstly over-enriched on the sheet structure; the excessive enriched atoms were then released with the dissolution of sheet structure; meanwhile, corrosion of nZVI proceeded and Co^2+^ was surface reduced or migrated into the cavity structure, which provides the space for the reaction of the contaminants with the nZVI core. This migration also demonstrates how the evolved cavity structure can be used to potentially utilize the iron core and rise to a higher removal capacity.

### System pH Control Removal

**Figure 6:**
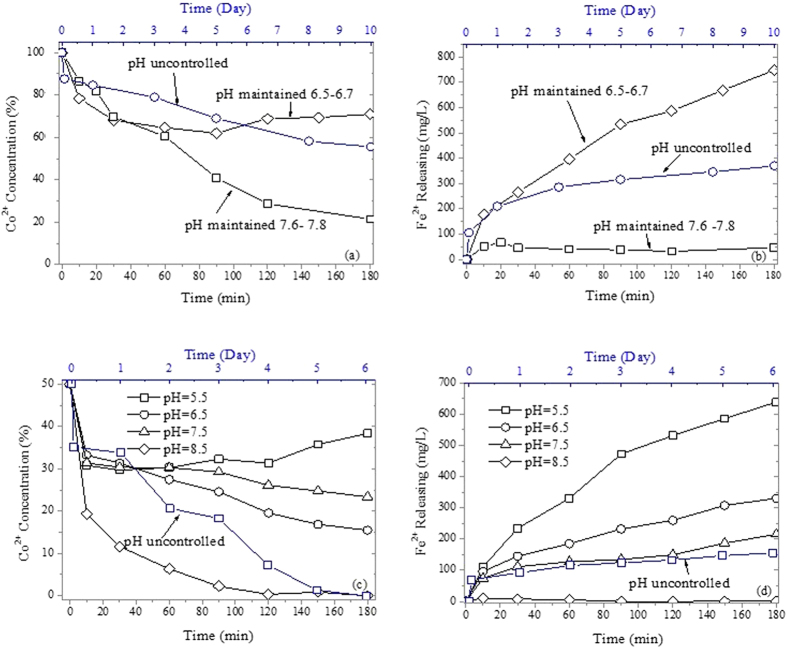
Effect of system pH on removal kinetics and Fe^2+^ release at initial Co^2+^ concentrations of 1000 mg/L (a,b) and 50 mg/L (c,d).

Removal experiments controlled by the system pH were conducted to further investigate the influence of system pH, as shown in Fig. 6. It should be noted that it is difficult to control a too high or too low system pH for the 1000 mg/L Co^2+^ solution and the pH fluctuation was greater because of the significant influence of the liquid phase. The higher system pH is beneficial for the formation of the sheet structure (Figure S20a, Figure S21), and accelerated the removal because Fe^2+^ and Co^2+^ formed hydroxide coprecipitates in high system pH.

Most previous studies reported that lower initial pH decreases the removal of metal cation by nZVI because a lower pH increases the surface potential of nZVI, leading to cation desorption^1^,^2^,^3^,^18^. However, the results could be different if the removal is not exactly sorption. Removal in a lower system pH was more efficient than that in uncontrolled system pH; also, removal was more efficient for the system pH of 6.5 than for the system pH of 7.5. However, too low system pH is unfavorable for removal, leading to excessive corrosion of nZVI (Fig. 6b,d) that results in the release of Co^2+^. As mentioned above, the sheet structure would prevent the further reaction between the iron core and Co^2+^ solution while the cavity structure could develop further reactions between the iron core and Co^2+^ solution; therefore, a subacidity condition, which could accelerate the sheet structure dissolution and cavity structure formation, would be beneficial for removal. The XPS analysis (Figure S23) also demonstrated the accelerated reduction of Co^2+^ at low system pH.

### Structure Pre-controlled Removal

**Figure 7:**
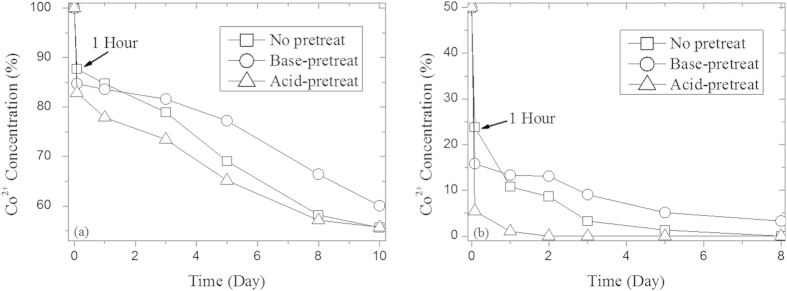
Effeect of structure pre-control on the removal at initial Co^2+^ concentration of 1000 mg/L (a) and 50 mg/L (b).

NZVI particles were pre-controlled to form the sheet wrapped core-shell and cavity structures by base-pretreatment and acid-pretreatment (Figure S24 a and i). The results of 1 g/L pretreated particles reacting with 1000 and 50 mg/L of Co^2+^ are shown in Fig. 7: acid-pretreated cavity particles were more efficient than the not pretreated nZVI; however, base-pretreated sheet wrapped particles were less efficient than the not pretreated nZVI. As shown in Figure S24, the evolution of nZVI is accelerated and delayed by acid-pretreatment and base-pretreatment, respectively: the structure of acid-pretreatment particles after reaction for 3 days exhibited sheet wrapped cavity structures similar to that of the not pretreated particles after 5 days’ and base-pretreated particles after 8 days’ in 1000 mg/L of Co^2+^ solution. In other words, the structural evolution influenced the removal process and mechanism: acid-pretreatment boosted the dissolution of the sheet structure and formed more cavities leading to a more efficient removal; base-pretreatment formed more sheet structure, preventing further reaction and necessitating more time for evolution to the cavity structure.

## Conclusions

As discussed above, the structure of nZVI evolves and different evolved structures could have distinct effects on the removal performance and mechanism. These structural evolutions could change the characteristics of the nZVI surface and bulk and alter the subsequent reactions. It is important to discuss the “structural influence” of the other evolved structure on the reaction process because in the actual reaction nZVI does not exist in the core-shell structure. Furthermore, if we ignore the factors that influence the structural evolution, it would appear that completely different results are obtained even for similar reaction conditions; these differences are difficult to explain if the influence of the structural changes is ignored. For example, controversial results were obtained in two previous studies focused on the reduction of Ni^2+^. Li and Zhang reported that approximately 65% of Ni^2+^ was reduced by 5 g/L nZVI after reacting for 3 hours with 1000 mg/L of Ni^2+^^11^,^23^. However, Efeca *et al.*^22^ reported that no Ni^2+^ was reduced to zero valence by 2.5 ~ 10 g/L nZVI after reacting for 4 hours with 500 mg/L of Ni^2+^. Thus, completely opposite results were obtained. In fact, Efeca obtained an extensively oxidized and “needle-like” structure very similar to the sheet structure in our study (Figure S21) which could prevent electron transfer and reduction. Efeca *et al.*^22^ attributed the contradiction to that high Ni^2+^ concentration tending to form surface precipitation; however, the excessive precipitation cannot be attributed to the high cation concentration because Li and Zhang used much higher Ni^2+^concentration. The most probable origin of the disagreement could rather be related to excessive oxygen introduced to the reaction due to the 80%-empty reaction tube as described in their paper^22^. Moreover, Li and Zhang reported that Ni^2+^ was released back to solution in 3 hours^23^, which could be regarded as the result of the dissolution of surface structure just as described above (Figs 4 and 5). In fact, higher Ni^2+^ concentration would enhance the structural evolutions due to its lower system pH.

## Methods

### Reagents

All the chemical reagents used in this study, such as FeCl_3_, NaBH_4_, CoCl_2_•6H_2_O, C_2_H_6_O, NaOH, HCl, HNO_3_, were reagent grade obtained from China Chemical Company. All the solutions used in this study were prepared in ultra-pure water.

### Preparation of nZVI

nZVI particles were synthesized using the method of liquid-phase reduction of ferric trichloride by sodium borohydride as described elsewhere^30^,^31^. In brief, the NaBH_4_ (0.25 M) solution was dropped at the rate of 0.25 mL/s into the FeCl_3_ (0.05 M) solution with the 1:1 volume ratio. The iron particles were separated from the solution using vacuum filtration and then washed three times with ethanol to prevent immediate oxidation. The nanoparticles were stored in ethanol, and mass fraction of the turbid liquid was approximately 40 ~ 50%.

### Removal Experiments

The removal of Co^2+^ was investigated in long term of 10 days and short term of 1 hour, respectively. The experiments were conducted in 50 mL polypropylene centrifuge tubes that were shaken in a shaker (200 rpm) at 25 °C under anoxic condition. In each separate experiment, 0.05 g nZVI was added into 50 mL Co^2+^ solutions with initial concentrations of 50, 500, 1000 mg/L. The experimental conditions were chosen for comparison to previous work (Support Information Section 1).

### Effect of System pH Control on Removal

The effect of system pH (measured in the mixture of nZVI and solution) control on Co^2+^ removal was investigated at system pH values of 8.5, 7.5, 6.5 and 5.5 in 50 mg/L Co^2+^ anoxic solution and of 7.6–7.8 and 6.5–6.7 in 1000 mg/L Co^2+^ anoxic solution, respectively. The system pH was controlled by an online pH controller (HACH Sc200) during the reactions. Nitrogen flow was used to keep the solution anoxic. HCl and NaOH were used for pH control to eliminate the disturbance of other ions. The nZVI dose was 1 g/L and the reaction took place for 180 min.

### Structure Pre-Control of nZVI

nZVI particles stirred in NaOH solution maintaining the system pH at 8.5 for 3 hours were used as the base pre-treatment sample and those stirred in HCl solution maintaining the system pH at 6.5 for 3 hours were used as the acid pre-treatment sample. The operating conditions were the same as those described for system pH control removal. After pre-treatment, the particles were centrifuged and immediately used for further reaction as mentioned in removal experiments, and the nZVI dose at 1 g/L was re-calculated after pretreatment.

### Metal Ions Concentration Measurement

The metal ions concentrations in solutions were determined with a PerkinElmer Optima 2100 DV inductively coupled plasma optical emission spectrometry (ICP-OES). Liquid samples were centrifuged at 6000 rpm for 3 minutes for solid-liquid separation and then acidified with 4% ultrahigh purity HNO_3_.

### Characterization

The microstructural evolution of nZVI particles was determined using high resolution transmission electron microscope (HR-TEM) images. HR-TEM analysis was performed on a JEOL JEM 2011 microscope with an INCA Energy Dispersive Spectrometer (EDS) system at 200 kV. The nZVI samples were dispersed in alcohol and then dropped on copper films dried at 50 °C for TEM analysis. The nZVI samples were also analyzed using scanning electron microscopy (SEM) with a Philips XL 30 microscope at 20 kV to characterize the structures. Prior to SEM analysis, the nZVI samples were firstly centrifuged and freeze-dried, then sprinkled onto glass analysis disks. X-ray photoelectron spectroscopy (XPS) of nZVI was analyzed to study the conversion of the element contents and valence states on nZVI surface. XPS analysis was performed on a Perkin Elmer PHI 5000 ESCA System with Al Kα radiation at 1486.6 eV. The solid samples were first centrifuged from solutions and then dried under vacuum at 25 °C for XPS analysis.

## Additional Information

**How to cite this article**: Zhang, Y. *et al.* Structural Evolution of Nanoscale Zero-Valent Iron (nZVI) in Anoxic Co^2+^ Soultion: Interactional Performance and Mechanism. *Sci. Rep.* **5**, 13966; doi: 10.1038/srep13966 (2015).

## Supplementary Material

Supplementary Information
