# Supplementary material for: Structural Evolution of Nanoscale Zero-Valent Iron (nZVI) in Anoxic Co2+ Solution: Interactional Performance and Mechanism
Source: Sci Rep. 2015 Sep 10;5:13966. doi: 10.1038/srep13966 (PMC4564818; doi:10.1038/srep13966)
Supplement: Supplementary Information [file srep13966-s1.pdf]

Structural Evolution of Nanoscale Zero-Valent Iron (nZVI) in Anoxic Co^{2+} Soultion: Interactional Performance and Mechanism

Yalei Zhang, †* Wen Chen,† Chaomeng Dai, ‡* Chuanlong Zhou,† Xuefei Zhou†

† State Key Laboratory of Pollution Control and Resources Reuse, Tongji University, Shanghai 200092, China.

‡ College of Civil Engineering, Tongji University, Shanghai 200092, China.

Correspondence and requests for materials should be addressed to Y.Z.(zhangyalei@tongji.edu.cn) or C.D.(daichaomeng@tongji.edu.cn)

Comparative Research.

Uzumet al. [1] have detailed investigated the performance and mechanism of nZVI reacted with Co^{2+}. They used $CoCl_2$ as Co source and liquid reduction for preparation of nZVI. The concentration of Co^{2+} were from 1 to 1000 mg/L, and they added 0.05g nZVI to 40 mL Co^{2+} solution, namely, the dosage of nZVI was 1.25 g/L. However, they did not report the sharking condition. Our experimental conditions were chosen based on this comparative research.

Uzum et al. [1] reported that all the removal equilibrium of Co^{2+} by nZVI was achieved at about 30 min in a period of 2 hours, and the removal capacity was up to 172 mg/g. They also conducted some reactions for 24 hours to investigate pH variations; however, they did not report the removal results in a period of 24 hours.

Removal Results.

The compare of long term and short term removal kinetics with 1 g/L nZVI are presented in Figure S1. The short term removal kinetics were similar to the results of Uzum, rapid removal stopped in a period of 30 min [1]. However, the long term removal results show differences. The long term removal rates achieved100%, 74.7%, 45.2% at initial Co^{2+} concentration of 50 mg/L, 500 mg/L and 1000 mg/L, in a period of 10 days, respectively.

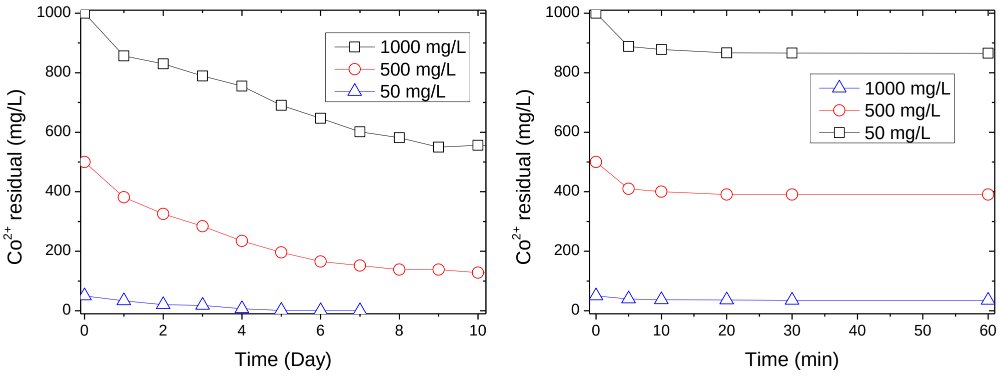

Figure S1. Comparison of removal kinetics between long term and short term experiments with 1g/L nZVI.

The compare of long term and short term removal capacity with 1 g/L nZVI are presented in Figure S2. The highest capacity of long term is up to 452.2 mg/g, which is tripled to the highest capacity of short term (148.8 mg/L). Also the short term removal capacity is similar to the previous research (172 mg/g) [1].

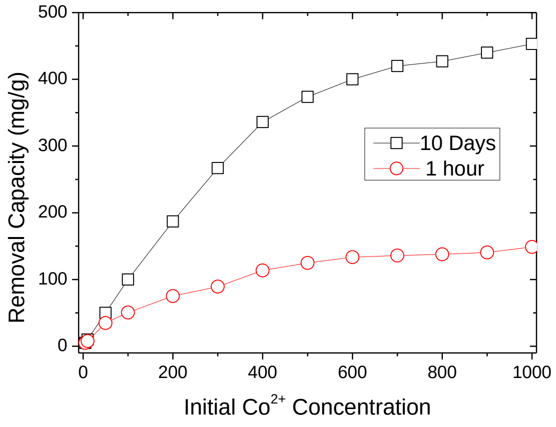

Figure S2. Comparison of removal capacity between long term and short term experiments with 1g/L nZVI.

The compare of long term and short term Fe^{2+} releasing with 1 g/L nZVI are presented in Figure S3. Higher initial Co^{2+} concentration resulted in more Fe^{2+} releasing; Fe^{2+} releasing and Co^{2+} removal always have similar tendency, Fe^{2+} continuously released when the long term removal lasted, and kept invariant when the rapid short term removal stopped. The excessive Fe^{2+} releasing may attribute to the further reaction between Co^{2+} and nZVI, as Li and Zhang reported, Fe^{2+} will release when Ni^{2+} reduced by nZVI [2].

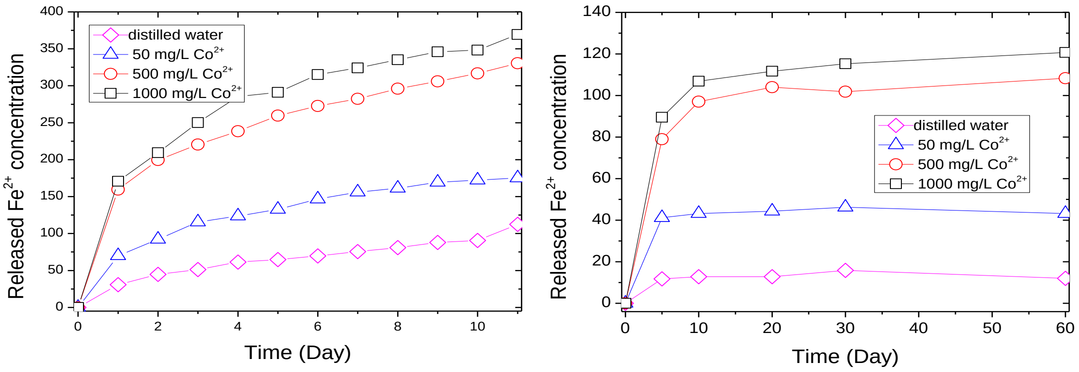

Figure S3. Comparison of Fe^{2+} releasing between long term to short term experiments with 1g/L nZVI.

Desorption experiments were also conducted. The samples after various removal times were desorbing at 50 ml ultra-pure water for 2 hours. As shown in Figure S4, the higher initial Co^{2+} concentration may lead to more desorption, however, both the quantity and the ratio of desorption were decrease with reaction proceed, indicating more stable combination of Co^{2+} and nZVI.

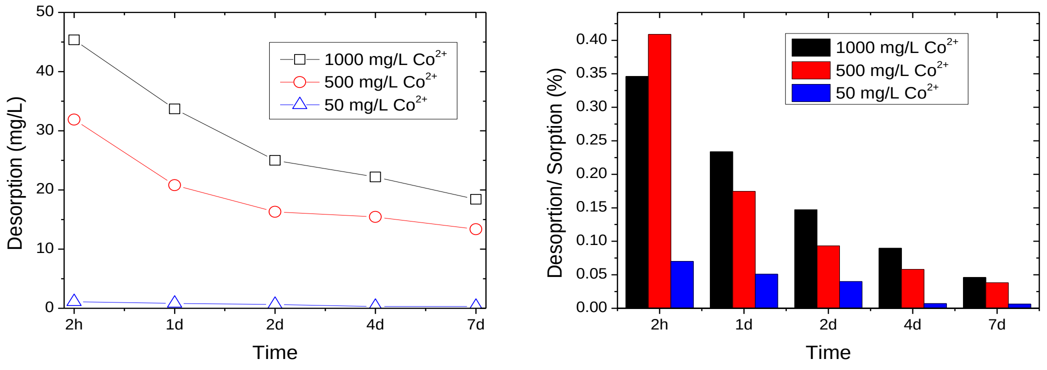

Figure S4. Desorption results of samples after various removal times.

Kinetics Fitting.

The pseudo first-order and pseudo second-order model were used for removal kinetics fitting. It should be noticed that it is not precise for using sorption kinetics models for this research because the reaction between nZVI and Co^{2+} is not exactly sorption. However, the kinetics models could indicate the tendency of the reactions to exam whether the short term kinetics could accord with long term kinetics.

The pseudo first-order equation (Lagergren's equation) describes adsorption in solid–liquid systems based on the sorption capacity of solids [3]. It is assumed that one cobalt ion is sobbed onto one sorption site on the n-ZVI surface:

$$A + Co^{2+}(aq) \xrightarrow{k1} ACo(\text{solid phase}) \quad (1)$$

Where A represents an unoccupied sorption site on the n-ZVI and k1 is the pseudo first order rate constant (h^{-1}). The linear form of pseudo first order model is:

$$\log(q_e - q_t) = \log q_e - \frac{k1}{2.302} t \quad (2)$$

Where qe and qt (mg/g) are the adsorption capacities at equilibrium and at time t (h), respectively.

Where k_2 is the rate constant for pseudo second-order adsorption ($g \bullet mg^{-1}h^{-1}$) and k_2qe^2 ($mg \bullet g^{-1}h^{-1}$) is the initial adsorption rate. This model assumed that one cobalt ion is sobbed onto two sorption site on the n-ZVI surface:

$$2A + Co^{2+}(aq) \xrightarrow{k2} A_2Co(\text{solid phase}) \quad (3)$$

The pseudo second-order rate expression, which has been applied for analyzing chemisorption kinetics from liquid solutions [4-5], the linear form is:

$$\frac{t}{qt} = \frac{1}{k_2 q_e^2} + \frac{1}{q_e} t \quad (4)$$

The result of Kinetic model fitting is shown in Table S1. The high r^2 values of short term pseudo second-order fitting indicating that the removal of Co^{2+} by nZVI perfectly fellow a second-order sorption model in short term reaction. However, the low r^2 values of long term fitting indicates that long term removal kinetics did not exactly fellow either sorption model because the long term removal including many other reactions beside sorption.

Table S1. Kinetic Model Fitting Results

Co^{2+} concentration (mg/L)	Pseudo first-order			Pseudo second-order		
	R^2	qe (mg/g)	K_1	R^2	qe (mg/g)	K_2
Short Term (Time: minutes)						
50	0.799	8.16	0.082	0.999	15.625	0.027
500	0.930	60.81	0.138	0.999	125.76	0.0063
1000	0.900	70.63	0.163	0.999	142.85	0.0082
Long Term (Time: hours)						

50	0.870	69.34	0.025	0.953	83.33	1.25×10-4
500	0.965	462.38	0.016	0.989	520.83	2.15×10-5
1000	0.926	533.33	0.014	0.893	729.92	9.10×10-6

However, we could found distinct difference between short term and long term kinetics. The short term reaction kinetics did not follow the long term reaction equations; the removal amounts in short term reactions are quite higher than the values which were calculated by the long term equations: 1) theoretical removal values calculated by long term pseudo first-order equations were 1.73, 9.48, 7.312 mg/g for 50, 500, 1000 mg/L Co^{2+}; 2) theoretical removal values calculated by long term pseudo second-order equations were 0.87,5.76,4.81 mg/g for 50, 500, 1000 mg/L Co^{2+}; 3) experimental values 14.88, 109.57, 134.23 mg/g. The results indicate that rapid and efficient equilibriums had been achieved in short term reactions, however then, altered by long term reactions. As discussed in manuscript Sheet Structure, the short term equilibriums were caused by the rapid formation of sheet structure and then altered by the dissolution of sheet structure in long term reaction.

Evolution Images

These additional images (Figure S5- Figure S11) provide an integrate vision of nZVI structural evolutions in various conditions. It should be noticed that, in Figure S11, hollows on the particles in red circles were obtained by SEM, which just according with the TEM images, indicating that the cavity should be bowl shaped.

As discussed in manuscript Sheet Structure, system pH only implies the macroscopical pH influenced by both phases. Actually, solid phase may have more influence to the interface, so sheet structure could still be slightly determined at very few part of nZVI at higher magnification (Figure S12).

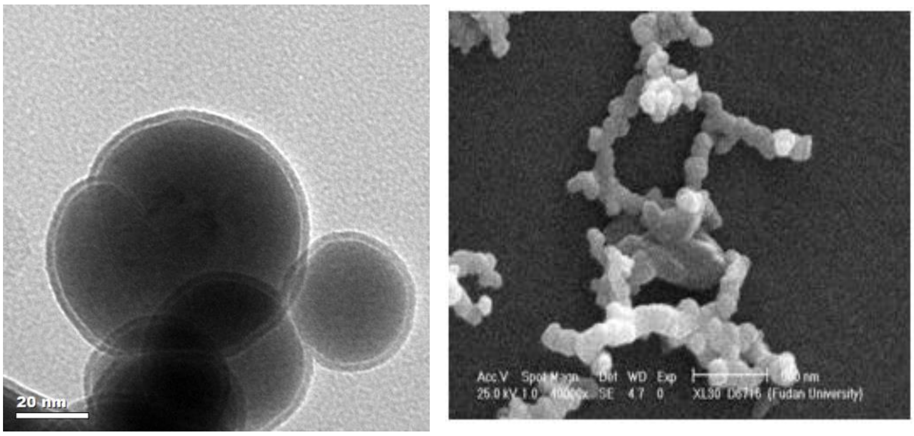

Figure S5. Typical TEM and SEM images of chain-like nZVI.

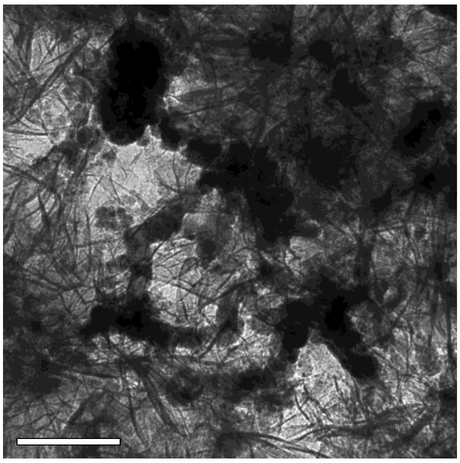

Figure S6. Typical TEM images of sphere surrounded chain-like nZVI after reacting with 50 mg/L Co^{2+} for 1 hour.

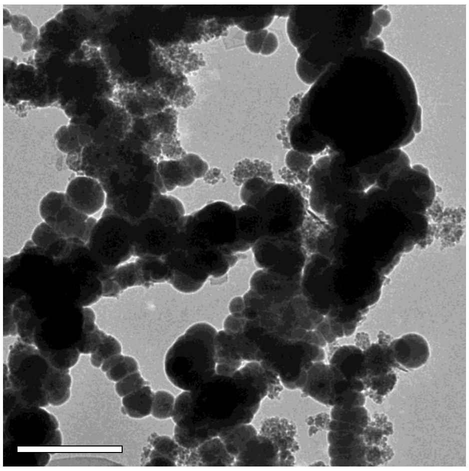

Figure S7. Typical TEM images of sphere surrounded chain-like nZVI after reacting with 50 mg/L Co^{2+} for 1 day.

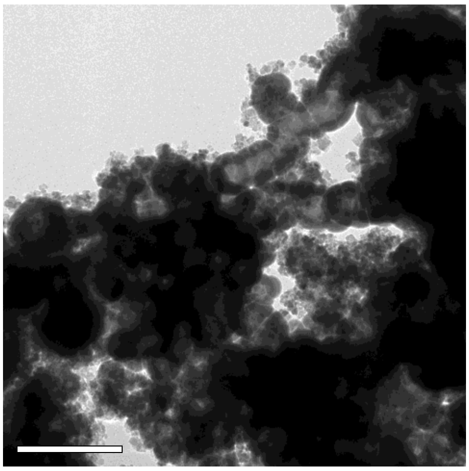

Figure S8. Typical TEM images of sphere surrounded cavity after reacting with 50 mg/L Co^{2+} for 10 day.

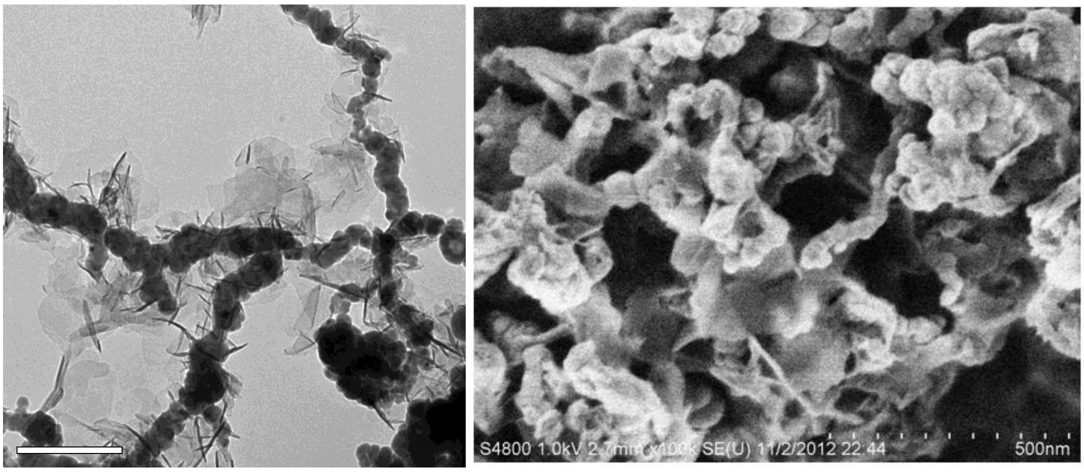

Figure S9. Typical TEM and SEM images of sheet wrapped chain-like nZVI after reacting with 1000 mg/L Co^{2+} for 1 hour.

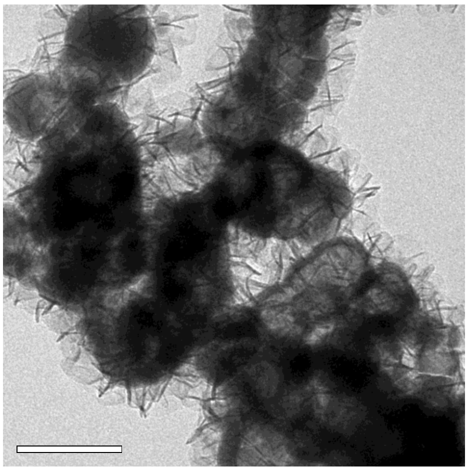

Figure S10. Typical TEM images of sheet warped cavity after reacting with 1000 mg/L Co^{2+} for 5 days.

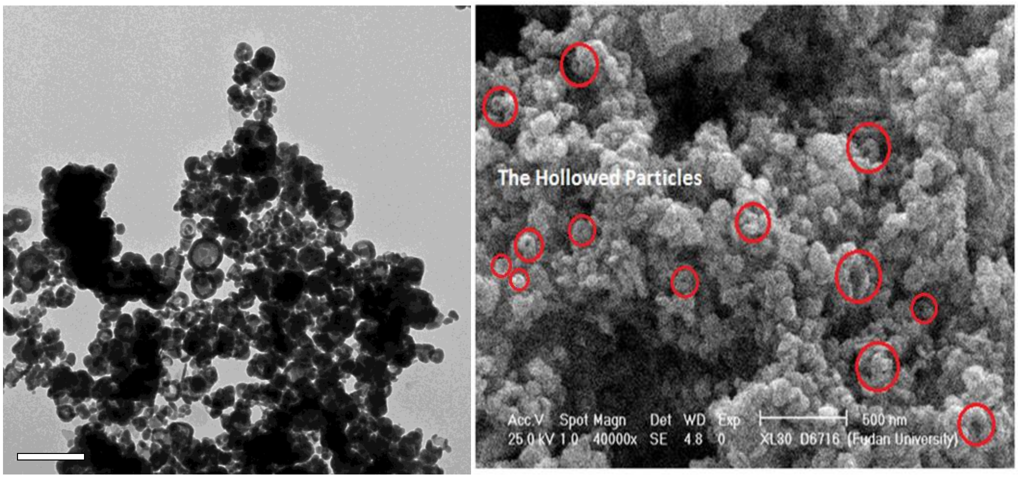

Figure S11. Typical TEM and SEM images of cavity structure after reacting with 1000 mg/L Co^{2+} for 10 days.

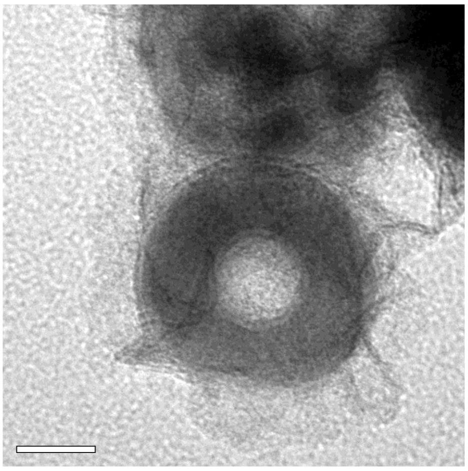

Figure S12. TEM images of little sheet wrapped cavity after reacting with 1000 mg/L Co^{2+} for 10 days.

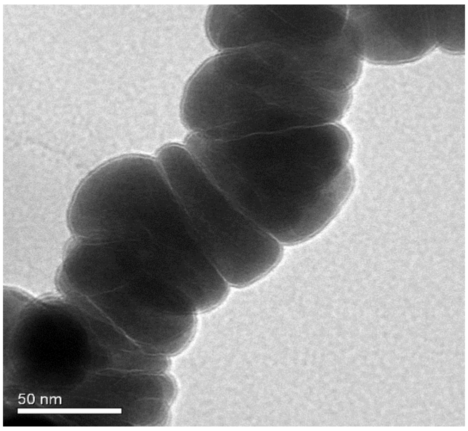

Figure S13. Typical TEM images of chain-like nZVI after reacting with deionized water for 1 hour.

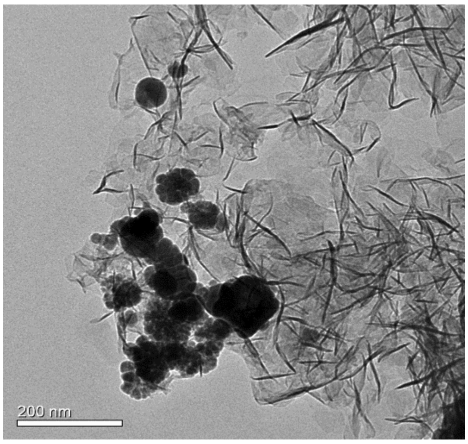

Figure S14. Typical TEM images of sheet warped chain-like nZVI after reacting with deionized water for 5 days.

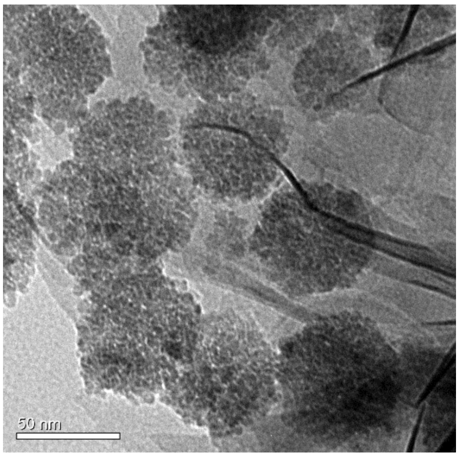

Figure S15. Typical TEM images of sphere structure after reacting with deionized water for 10 days.

EDS Analysis

The results of EDS analysis is shown in Figure S16. The ratio of Fe to O on core structure is 2:1, indicating that the ratio of Fe metal to Fe oxides may be 5:1. And the ratio of Fe to Co to O on cavity structure is 3.5:1:1.5 also showing a dominance of zero valent metal. The ratio of metal to O on sheet structure is approximate to 1:2, indicating a dominance of metal hydroxides or metal oxides. However, Co could be hardly determined on sphere structure, which indicated that sphere was an iron oxide or hydroxide. It should be noticed that the actual quantity of atoms cannot be reflect by the EDS analysis due to the material density of different parts maybe different. That is, Co atom enrichment may be quite different although the ratio of Co on sheet and cavity is approximate.

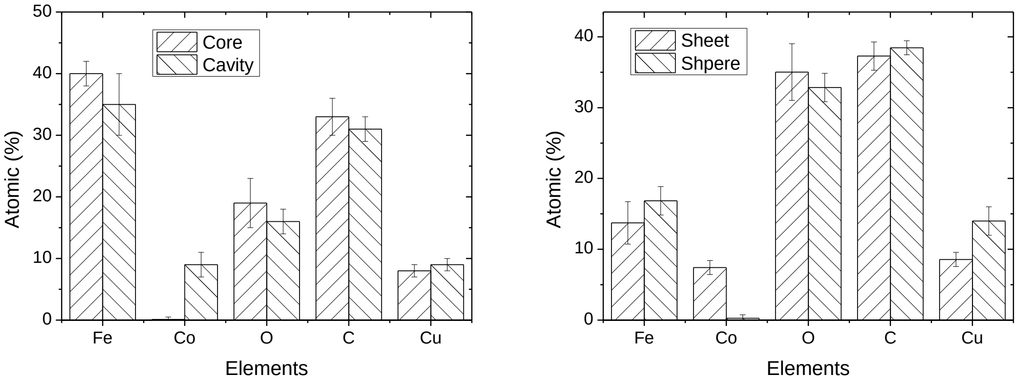

Figure S16. Atomic concentrations of different elements on iron core, cavity, sheet and sphere structures.

XPS analysis

The results of XPS analysis of Fe 2p of nZVI reacted with 1000 mg/L Co^{2+} at different times are presented in Figure S17. As the results shown, a small quantity of zero valent iron with the Fe 2p3/2 line centered at 707.0 $\pm$ 0.1 eV binding energy and the Fe 2p 1/2 line centered at 720.1 $\pm$ 0.1 eV and a large quantity of ferric iron with the Fe 2p3/2 line centered at 711.6 $\pm$ 0.1 eV binding energy and the Fe 2p 1/2 line centered at 724.6 $\pm$ 0.1 eV could be determined on fresh nZVI surface due to the covering FeOOH shell structure leading few exposure of zero valent iron [6-9]. Accordingly, zero valent iron could be scarcely determined on the sheet wrapped structure which formed after 1 hour reacting. However, the quantity of the zero valent iron on particle surface obviously increased with the diminishment of the sheet structures. After 10 days reacting, the proportions of zero valent iron on the pure cavity structures even

surpassed the fresh nZVI. That is, the surface zero valent iron was re-exposed with the dissolution of the sheet structures.

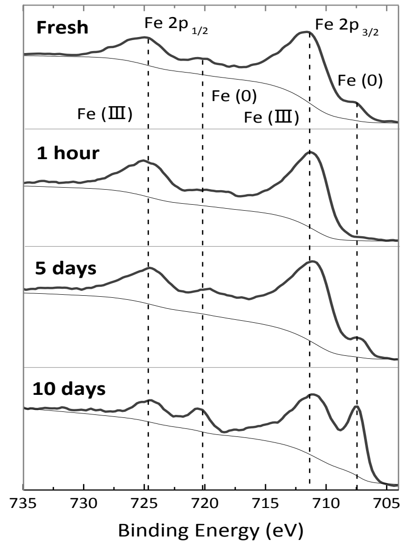

Figure S17. XPS spectra of Fe 2p of nZVI reacting with 1000 mg/L Co^{2+} at different times.

The results of XPS spectra of Co 2p 3/2 of nZVI surface are presented in Figure S18: only Co (II) can be determined on nZVI surface after 1 hour, which was in accordance with Uzum [1]; about 10% Co (II) was reduced by nZVI to Co (0) after 5 days reacting, and finally about 52% Co (0) was formed after 10 days reacting, the photoelectron peaks at 786.2, 782.6 and 778.1 eV are assigned to the 2p 3/2 binding energies of Fe auger, Co (II) and Co (0), respectively [11-13]. Obviously, a further reduction could be determined by XPS analysis. The association between the portion of zero valent iron and the reduction of Co (II) on nZVI surface could be prefect established, which means the quantity of zero valent iron on nZVI surface played a decisive role to the reduction of Co (II).

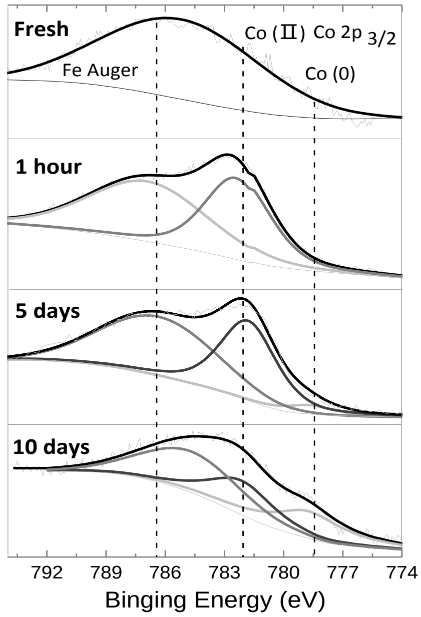

Figure S18. XPS spectra of Co 2p 3/2 of nZVI surface reacting with 1000 mg/L Co^{2+} at different times.

The results of XPS spectra of O 1s of nZVI surface are presented in Figure S19. Oxygen formed H_2O with the O 1s line centered at 532.9±0.1 eV binding energy [14], formed both iron and cobalt oxides with the O 1s line centered at 530.0±0.1 eV binding energy, formed FeOOH with the O 1s line centered at 531.4±0.1 eV binding energy [6-9]. However, the peak centered at 532.0±0.1 eV of this study cannot be determined by pervious researches, it may be the peak of Fe / Co coprecipitation due to the peak excursion. On fresh nZVI, FeOOH and iron oxides may be the dominance, then converted to the Fe / Co coprecipitation and oxides due to the formation of the sheet structure, finally back to FeOOH due to the dissolution of the sheet structure. On the other hand, the ratio of OH^- and O^{2-} to H_2O decreased with the reaction proceed indicating an increase of zero valent metal, which is according with the results of XPS analysis of Fe and Co.

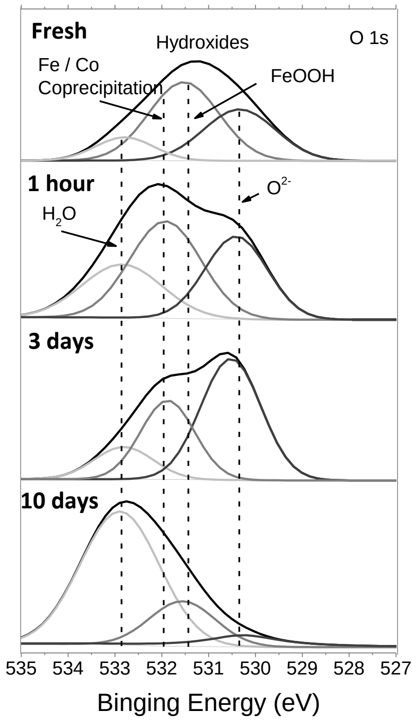

Figure S19. XPS spectra of O 1s of nZVI surface reacting with 1000 mg/L Co^{2+} at different times.

Precipitation pH

The theoretical pH leading the dissolution of the precipitation was deduced by the solubility product as equation (5) (6), supposing all precipitation was bivalent metal hydroxide as a simplification:

$$Ksp= [M^{2+}]\,[OH^-]^2 \quad (5)$$

$$pHp= 14+\log\sqrt{\frac{Ksp}{[M^{2+}]}} \quad (6)$$

Where Ksp, $[M^{2+}]$, $[OH^-]$ stands for solubility product constant, molarity of M^{2+}, molarity of OH^-, respectively. The Ksp of $Fe(OH)_2$ and $Co(OH)_2$ is 4.87×10^{-17} and 5.92×10^{-15} at 25 ℃, respectively [10].

The theoretical pH leading the dissolution of the $Fe(OH)_2$ and $Co(OH)_2$ at different times are calculated in Table S2.

Table S2. The calculation of the theoretical pH leading to the dissolution of $Fe(OH)_2$ and $Co(OH)_2$

Time (Day)	Fe^{2+} in 1000 mg/L (mg/L)	Dissolution pH	Fe^{2+} in 50 mg/L (mg/L)	Dissolution pH	Co^{2+} in 1000 mg/L (mg/L)	Dissolution pH	Co^{2+} in 1000 mg/L (mg/L)	Dissolution pH
0	0	/	0	/	1000	7.77	50	8.42
1 hour	170.84	7.10	69.85	7.30	856.62	7.81	33.77	8.51
1	209.43	7.06	92.13	7.24	830.01	7.81	20.66	8.61
2	250.05	7.02	115.68	7.19	789.17	7.82	18.27	8.64
3	285.53	6.99	123.68	7.17	754.74	7.83	7.24	8.84
4	291.05	6.99	132.74	7.16	690.50	7.85	1.33	9.21
5	315.20	6.97	146.74	7.13	646.82	7.87	0	/
6	324.23	6.96	156.05	7.12	601.44	7.88	0	/
7	335.12	6.96	161.23	7.11	581.86	7.89	0	/
8	345.93	6.95	169.36	7.10	549.59	7.90	0	/

9	348.36	6.95	172.26	7.10	555.95	7.90	0	/
10	369.41	6.93	175.20	7.10	547.81	7.90	0	/

System pH Controlled Experiments

The TEM images of structural evolution of nZVI in system pH controlled removal are presented as follow. The TEM images of structural evolution of nZVI in system pH controlled removal of 50 mg/L Co^{2+} are present in Figure S20. In 50 mg/L Co^{2+}, the sheet dissolved in pH of 6.5 was earlier than which in pH of 7.5 (Figure S20 b and c). The TEM images of structural evolution of nZVI in system pH at 7.6-7.8 of 1000 mg/L Co^{2+} are present in Figure S21. The TEM images of structural evolution of nZVI in system pH at 6.5-6.7 of 1000 mg/L Co^{2+} are present in Figure S22. As Figure S21 shown, the sheet structure would grow bigger with the removal process in high system pH and high initial Co^{2+} concentration. As shown in Figure S22, the sheet structure may rapidly dissolved and form cavity structure in a lower system pH, even it would take several days' evolution in pH uncontrolled system.

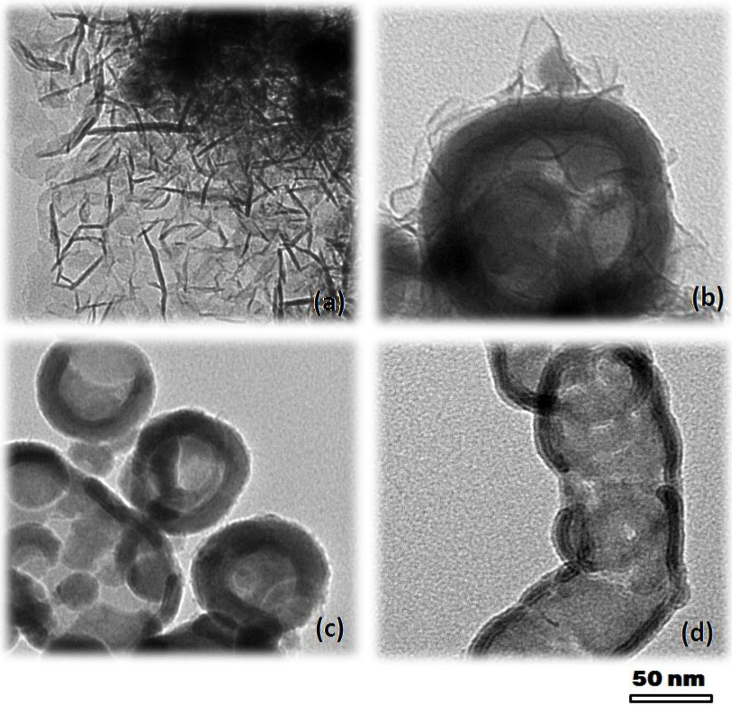

Figure S20. The typical TEM images of the nZVI evolution with 50 mg/L Co^{2+} in 3 hours at different system pH : (a) pH= 8.5, (b) pH=7.5, (c) pH=6.5, (d) pH=5.5.

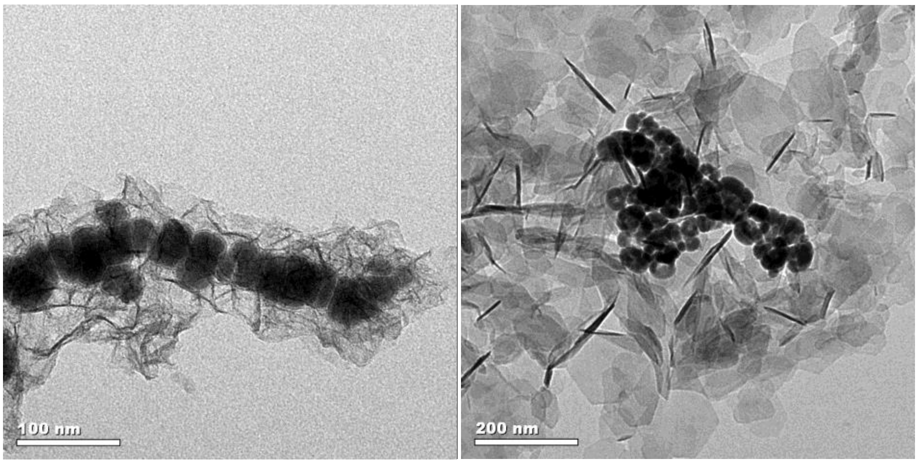

Figure S21. TEM images of nZVI in 1000 mg/L Co^{2+} at system pH of 7.6-7.8 (left: 20 min, right: 180 min).

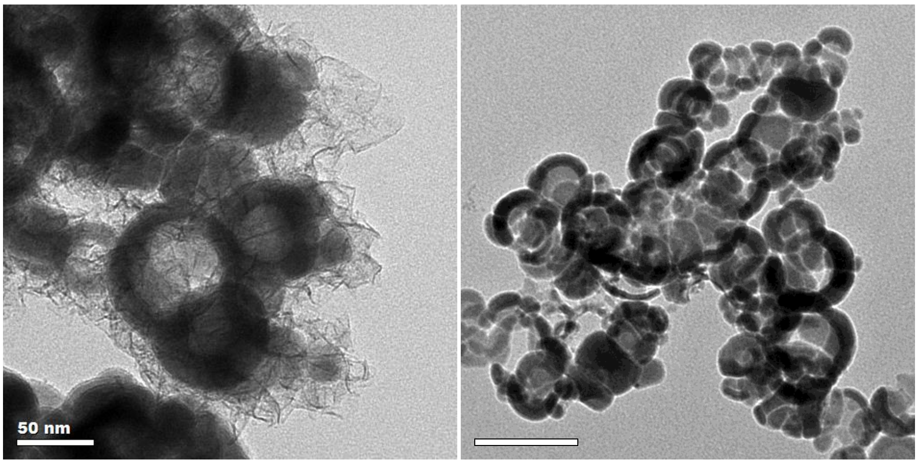

Figure S22. TEM images of nZVI in 1000 mg/L Co^{2+} at system pH of 6.5-6.7 (left: 20 min, right: 180 min).

The XPS analysis results of nZVI reacted 3 hours with 1000 mg/L Co^{2+} at different system pH are shown in Figure S23. In high system pH, Co mainly formed divalent, however, about 33% was reduced to zero valent in low system pH which achieved after 8 days in uncontrolled system.

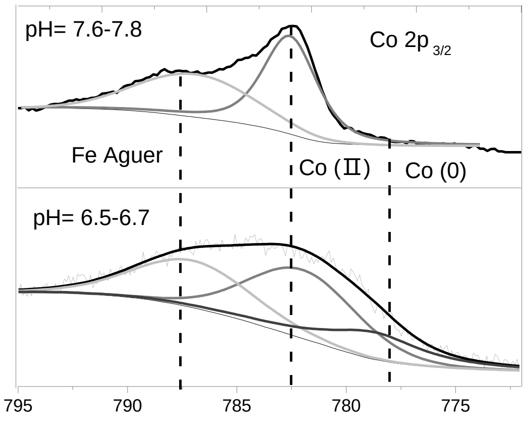

Figure S23. XPS spectra of Co 2p 3/2 of nZVI surface reacting with 1000 mg/L Co^{2}+ for 3hours at different system pH.

Structure Pre-control

The results of acid-pretreatment and base-pretreatment is present in Figure S24 a and i, cavity structure and sheet wrapping structure was perfectly obtained. And these pretreated particles were immediately used for further reaction. The structural evolution of pretreated particles in 1000 mg/L Co^{2+} were presented in Figure S24. The pretreatment change the stage of structural evolution: the structure of acid-pretreatment after 3 days' reaction is similar to the structure of no pretreated after 5 days' reaction, also similar to the structure of base-pretreatment after 8 days' reaction; the structure of acid-pretreatment after 5 days' reaction is similar to the structure of no pretreated after 10 days' reaction; the structure of no pretreated after 3 days' reaction is similar to the structure of base-pretreatment after 5 days' reaction.

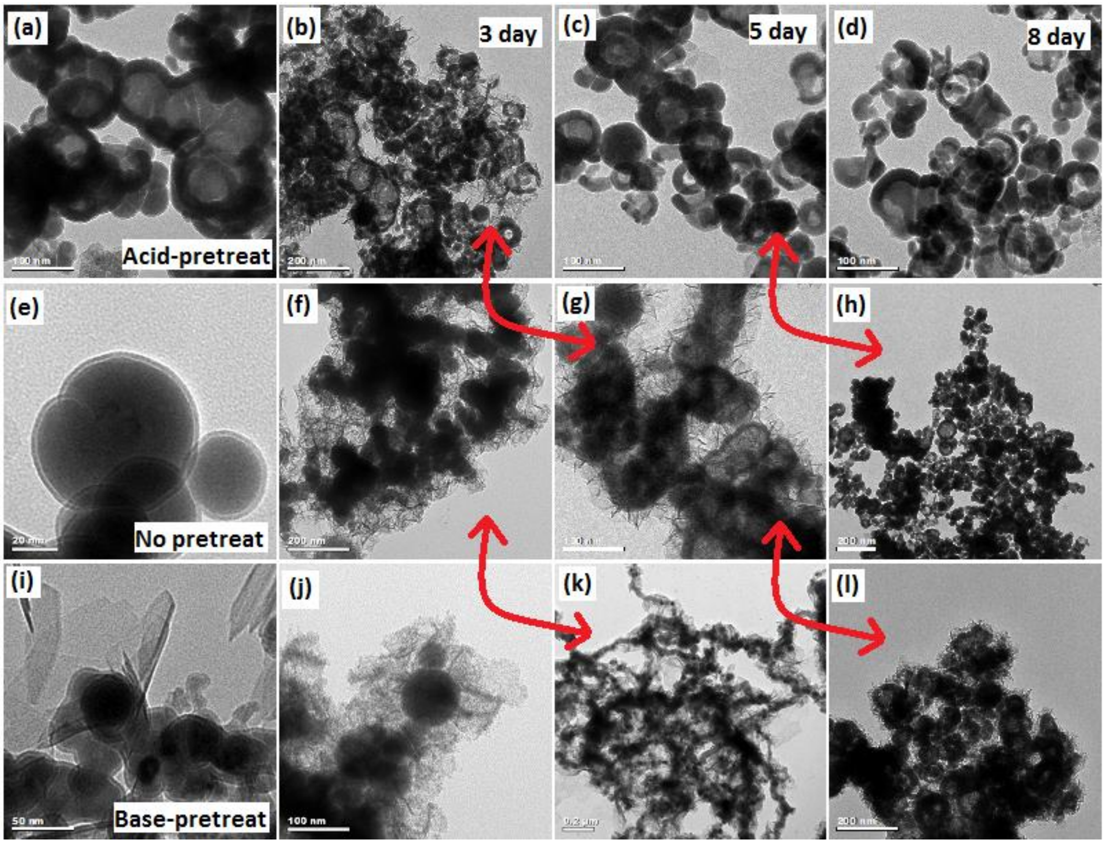

Figure S24. TEM images of the structural evolution of nZVI in reaction of 1000 mg/L Co^{2+} with acid-pretreatment (a-d), no pretreatment (e-h) and base-pretreatment (i-l).

XRD analysis and discussion

XRD analysis was carried out on a Bruker D8Advance X-ray diffraction instrument (Cu Kα), the diffraction angle (2θ) from 10 to 90 °was scanned. The samples were centrifuged and then vacuum drying at 40℃ or freezing drying at -10℃ for XRD analysis. The XRD analysis results are shown in Figure S25. The peak reveals the existence of iron on the basis of the Jade pdf-# 65-4899(Fe), the peaks at the 2θ of

44.9 °, 64.9 °, 82.2 ° indicated the presence of iron. However, the results were significantly influenced by the pretreatment of samples in this research, XRD analysis to the same sample with vacuum drying or freezing drying was different: no obvious peak could be found when he samples were freezing dried (Figure S25a); peaks could be distinct when he samples were vacuum drying at 40℃ (Figure S25b). Therefore, the prepared nZVI presented an amorphous phase of iron, and would not change to crystal in reactions. And heating of nZVI would change the amorphous phase iron to crystal as shown in Figure S25b.

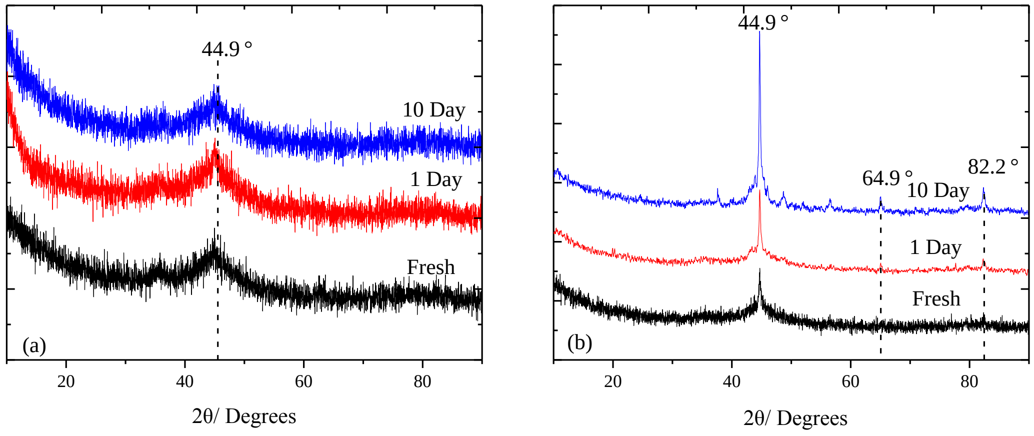

Figure S25. XRD analysis results of nZVI: a) samples were freezing dried; b) samples were vacuum dried.

REFERENCES

1. Uzum, C. et al. Application of zero-valent iron nanoparticles for the removal of aqueous Co^{2+} ions under various experimental conditions. *Chemical Engineering Journal* . **144**, 213-220(2008).
2. Li, X. Q. & Zhang, W. X. Iron nanoparticles: the core-shell structure and unique properties for Ni(II) sequestration. *Langmuir* . **22**, 4638-4642(2006).
3. Ho, Y. S. Citation review of Lagergren kinetic rate equation on adsorption reactions. *Scientometrics* . **59**, 171-177(2004).
4. Azizian, S. Kinetic models of sorption: a theoretical analysis. *J Colloid Interf Sci* . **276**, 47-52(2004).
5. Ho, Y. S. Review of second-order models for adsorption systems. *Journal of hazardous materials* . **136**, 681-689(2006).
6. Sun, Y. P., Li, X. Q., Cao, J. S., Zhang, W. X. & Wang, H. P. Characterization of zero-valent iron nanoparticles. *Advances in colloid and interface science* . **120**, 47-56(2006).
7. Grosvenor, A. P., Kobe, B. A. & McIntyre, N. S. Studies of the oxidation of iron by water vapour using X-ray photoelectron spectroscopy and QUASES (TM). *Surf Sci* . **572**, 217-227(2004).
8. Grosvenor, A. P., Kobe, B. A. & McIntyre, N. S. Studies of the oxidation of iron by air after being exposed to water vapour using angle-resolved X-ray photoelectron spectroscopy and QUASES. *Surf Interface Anal* . **36**, 1637-1641(2004).
9. Grosvenor, A. P., Kobe, B. A., Biesinger, M. C. & McIntyre, N. S. Investigation of multiplet splitting of Fe 2p XPS spectra and bonding in iron compounds. *Surf Interface Anal* . **36**, 1564-1574(2004).
10. Stumm, W. M. & J. J. Aquatic Chemistry, 3rd ed. Wiley & Sons: New York 1996.

11. N.S. McIntyre & G. Cook,rosvenor. X-ray photoelectron studies on some oxides and hydroxides of cobalt, nickel, and copper. *Anal. Chem.* **47**, 2208-2213(1975).

12. N.S. McIntyre, D.D. Johnston, L.L. Coatsworth, R.D. Davidson &J.R. Brown. X-ray photoelectron spectroscopic studies of thin film oxides of cobalt and molybdenum. *Surf. Interface Anal.* **15**, 265-272(1990).

13. B.J. Tan, K.J. Klabunde & P.M.A. Sherwood. XPS studies of solvated metal atom dispersed (SMAD) catalysts. Evidence for layered cobalt-manganese particles on alumina and silica. *J. Am. Chem. Soc.* **113**, 855-861(1991).

14. Efecan, N., Shahwan, T., Eroglu, A. E. & Lieberwirth, I. Characterization of the uptake of aqueous Ni^{2+} ions on nanoparticles of zero-valent iron (nZVI). *Desalination* . **249**, 1048-1054(2009).

Acknowledgements

This work was financially supported by the China Scholarship Council for Yiming Su, the National Key Technologies R&D Program of China (No.2012BAJ25B02).

Author contributions

Y.Z. , C.D. and W.C. wrote and corrected the manuscript text and figures, C.Z. prepared fig. S1-S12 and wrote the TEM measurement part, X.Z. prepared table.S1 and table.S2 and wrote XPS measurement part, W.C. prepared fig. 13-22 and wrote EDS measurement part. All authors reviewed the manuscript.

Additional information

Competing financial interests: The authors declare no competing financial interests.

†Electronic Supplementary Information (ESI) available: SEM images of chain-like nZVI；TEM images of sphere surrounded chain-like nZVI after reacting with 50 mg/L Co^{2+}； EDS analysis of nZVI; XPS spectra of Fe 2p of nZVI. See DOI: 10.1039/b000000x/
